# Enhancing Surface Icephobic Performance via Spontaneous Fracture Characteristics of Ice Crystals Regulated by Structural Scale Under Shear Flow Conditions

**DOI:** 10.1002/advs.78489

**Published:** 2026-10-25

**Authors:** Jiawei Jiang, Yizhou Shen, Yangjiangshan Xu, Junxian Xiang, Weilan Liu, Senyun Liu, Lingfeng Zhao, Jie Tao, Zhong Chen

**Affiliations:** ^1^ College of Materials Science and Technology Nanjing University of Aeronautics and Astronautics Nanjing People's Republic of China; ^2^ Beijing Blue Sky Innovation Center For Frontier Science Beijing People's Republic of China; ^3^ Institute of Advanced Materials Nanjing Tech University Nanjing People's Republic of China; ^4^ Key Laboratory of Icing and Anti/De‐icing China Aerodynamics Research and Development Center Mianyang People's Republic of China; ^5^ School of Materials Science and Engineering Nanyang Technological University Singapore Singapore

**Keywords:** Icephobic, shear flow, spontaneous fracture, structural scale, superhydrophobic

## Abstract

Icephobic surfaces are conventionally designed by minimizing ice adhesion; however, whether this strategy remains valid under aerodynamic shear remains poorly understood. Here, we reveal that an external flow field fundamentally alters ice adhesion dynamics by inducing the spontaneous fracture and detachment of ice crystals through aerodynamic shear forces. The ice‐substrate contact area is primarily determined by the initial droplet size prior to freezing, thereby governing the interfacial adhesion force. Furthermore, Ice detachment is controlled by a competitive interplay between the initial crystal size and its growth orientation, which together determine whether the aerodynamic loading is sufficient to overcome interfacial adhesion. Contrary to the conventional expectation that superhydrophobicity facilitates ice removal, the microstructural anchoring effect inherent to superhydrophobic surfaces impedes spontaneous ice detachment, resulting in substantially higher ice retention compared with conventional hydrophobic surfaces. By contrast, hydrophobic surfaces with nanoscale features exhibit a greater capacity to suppress runback‐ice accumulation under flow than conventional hierarchical superhydrophobic surfaces. These findings establish a mechanistic framework for flow‐induced spontaneous ice detachment and redefine an icephobicity design concept from minimizing adhesion toward controlling fracture dynamics.

## Graphical abstract

Aerodynamic shear induces spontaneous ice fracture and detachment on hydrophobic surfaces, governed by the interplay between initial ice‐crystal size, growth orientation, and interfacial adhesion. Unexpectedly, microstructural anchoring on superhydrophobic surfaces suppresses ice shedding. These findings reveal a flow‐dependent ice‐removal mechanism and highlight nanoscale hydrophobic surfaces as promising strategies for mitigating runback icing.

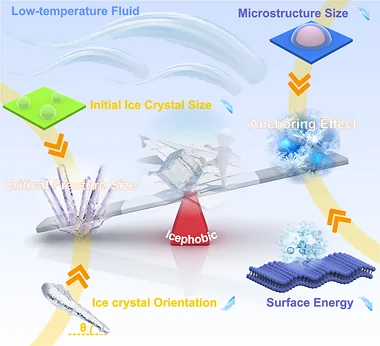

## 1 Introduction

Recently, unmanned aerial vehicles (UAVs) have witnessed rapid expansion across commercial, defense, and environmental monitoring applications, driving an increasing demand for reliable operation under diverse climatic and geographical conditions [1, 2, 3]. However, when UAVs encounter clouds containing supercooled water droplets, ice can readily accrete on exposed surfaces, particularly on aerodynamically critical components such as wings, substantially reducing flight performance and safety margins [4, 5, 6]. Flight experiments have demonstrated that icing can increase propeller power consumption by more than 80% and induce pronounced structural vibrations, thereby compromising flight stability and operational reliability. Consequently, icing remains a major obstacle to the reliable and large‐scale deployment of UAVs under adverse weather conditions [7, 8, 9, 10].

For UAVs with severely constrained onboard energy budgets, compared to conventional active anti‐icing approaches (including mechanical de‐icing [11, 12], electrical‐pulse de‐icing [13, 14], electrothermal de‐icing [15, 16, 17], and ultrasonic de‐icing [18, 19]), passive anti‐icing strategies with low energy requirements, lightweight and structural simplicity have emerged as a promising pathway for enhancing UAV icing resilience [20, 21, 22]. Therein, superhydrophobic materials have attracted considerable attention owing to their low droplet adhesion, ice nucleation delay and low ice adhesion, which can collectively mitigate ice accretion and facilitate ice removal [23, 24, 25].

Several pieces of evidence have demonstrated the effectiveness of superhydrophobic surfaces in delaying ice accretion and facilitating ice removal at the leading edge of aircraft wings [26, 27, 28, 29, 30]. However, for UAVs, icing is not confined to the leading edge. Supercooled microdroplets impacting the wing are always driven downstream by aerodynamic shear, and refreeze to form runback ice [31, 32, 33]. Runback ice ridges can substantially modify the aerodynamic profile, degrade aerodynamic performance, and compromise flight safety through blockage of flap gaps. For swept‐wing configurations, runback icing can additionally produce complex scallop‐shaped ice morphologies, further amplifying aerodynamic degradation [34]. Effective mitigation of the adverse effects of runback ice therefore remains an important challenge for the development of passive anti‐icing strategies for UAVs.

Although the efficacy of superhydrophobic surfaces in suppressing ice accretion at the leading edge has been widely demonstrated, whether this advantage can be extended to downstream runback ice formation remains poorly understood at the mechanistic level. Previous research generally assumed that ice‐shedding performance scales inversely with interfacial ice adhesion, such that weaker adhesion necessarily translates into more effective ice removal [35, 36]. Notably, runback icing is inherently a dynamic process sustained by aerodynamic shear [37]. The persistence and detachment of ice crystals under shear‐flow conditions depend not only on the magnitude of interfacial adhesion, but also on stress concentration and crack propagation within the solid‐ice contact region under tangential loading. Although the hierarchical micro‐nanostructure of superhydrophobic surfaces reduces the effective solid‐ice contact area, it simultaneously introduces numerous microscale asperities and geometric discontinuities which may impede the collective detachment of ice crystals.

Nowadays, a fundamental gap remains in understanding the anti‐icing performance of superhydrophobic surfaces under realistic aerodynamic conditions. Most studies have focused on ice adhesion strength and nucleation delay under static or quasi‐static loading, nevertheless, these conventional metrics do not incorporate the persistent aerodynamic shear that governs icing in an inflow environment [38, 39, 40]. Meanwhile, the limited numerical and experimental studies of runback icing have primarily addressed anti‐icing heat loads and the prediction of macroscopic ice morphologies [41, 42, 43]. Obviously, the surface microtopography of superhydrophobic surfaces has a non‐negligible impact on droplet migration, interfacial heat transfer, nucleation, and adhesion under sustained shear flow, fundamentally determining the form of runback ice. A systematic understanding of how surface microtopography regulates dynamic icing and de‐icing under continuous aerodynamic shear is essential for establishing physically informed design principles for icephobic surfaces.

In this work, dynamic icing experiments were performed on PTFE hydrophobic/superhydrophobic surfaces with different microstructural scales under shear flow conditions. The influence laws of environmental parameters and surface physical characteristics on the evolution of runback ice were systematically explored. The results reveal that aerodynamic shear can induce spontaneous fracture and detachment of ice crystals from the surface. Contrary to the conventional view that reduced ice adhesion necessarily enhances ice repellency, the interfacial anchoring introduced by the hierarchical micro‐nanostructure increases the critical stress required for ice detachment. Consequently, superhydrophobic surfaces with hierarchical structures exhibit lower dynamic ice‐repellent performance than hydrophobic surfaces bearing only nanostructures. This spontaneous ice repulsion theoretical model induced by the external wind field is expected to provide a mechanistic basis for the region‐specific design of icephobic surfaces for UAVs.

## 2 Results and Discussion

### 2.1 Fabrication and Characterization of PTFE Surfaces With Various Structural Scales

To investigate the icing behavior of various hydrophobic surfaces under shear flow, hydrophobic/superhydrophobic surfaces with systematically varied structural scales are fabricated by a simple spray‐coating process using low‐surface‐energy PTFE nanoparticles. By varying the PTFE content, the resulting surfaces are denoted PTFE‐0, PTFE‐3, PTFE‐5, PTFE‐10, and PTFE‐15. Scanning electron microscopy (SEM) revealed a progressive transition in surface morphology with increasing PTFE content (Figure 1a). The PTFE‐0 surface lacks distinct microscale clusters and is dominated by nanostructures consisting of strip‐like features approximately 330 nm in width. This relatively simple topography provides insufficient air entrapment to sustain a stable Cassie‐Baxter state, resulting in a water contact angle of 125.13°. With increasing PTFE content, petal‐like microstructures with characteristic dimensions of 10–15 µm emerge on the PTFE‐3 surface, exhibiting superhydrophobic characteristics with a contact angle of 154.37°. Further increases in PTFE content promote the growth and interconnection of microstructures with their characteristic size increasing from 17 µm for PTFE‐5 to 32 µm for PTFE‐15. The progressively enlarged hierarchical structures are accompanied by a monotonic increase in water contact angle, from 159.03° (PTFE‐5) to 166.84°(PTFE‐15). Consistent with the SEM observations, laser confocal microscopy shows that the maximum structural height of PTFE‐0 is only 3.1 µm, corresponding to a comparatively smooth hydrophobic surface (Figure 1b). As the microstructural scale ascends, the surface topography becomes progressively deeper, with the characteristic structural depth increasing from 75 µm (PTFE‐3) to 123 µm (PTFE‐15).

**FIGURE 1:**
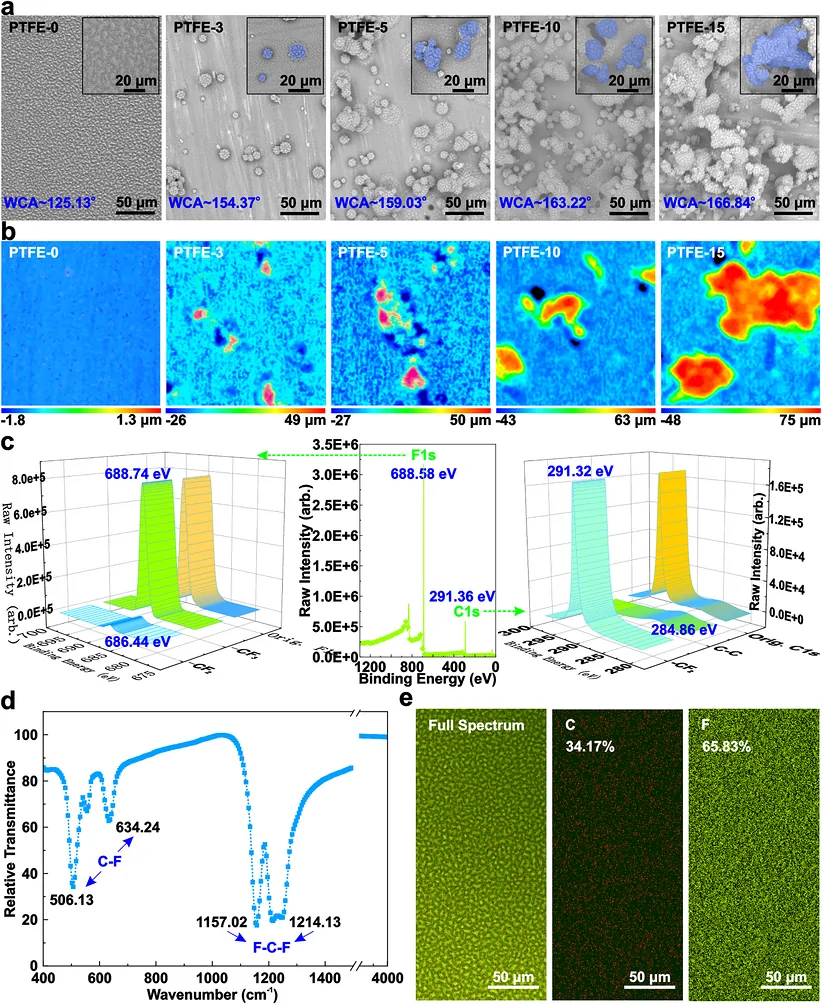
Morphology and composition analysis of different PTFE surfaces. (a) SEM morphology of five different PTFE surfaces. (b) Three‐dimensional contour images of five different PTFE surfaces. (c) XPS analysis of typical PTFE‐0 surface (C1s and F1s spectra are shown by green arrows, respectively). (d) FTIR curve of typical PTFE‐0 surface. (e) EDS image of typical PTFE‐0 surface.

Considering that a non‐ionic surfactant is introduced during the dispersion of the PTFE nanoparticles, X‐ray photoelectron spectroscopy (XPS) is performed to verify the chemical composition of the resulting surfaces and exclude residual contaminants (Figure 1c). It can be observed that the XPS survey spectrum contained only C and F signals. In the F1s spectrum, the peaks at 686.44 and 688.74 eV are assigned to C─CF and CF_3_ species, respectively, whereas the C1s peaks at 284.86 and 291.32 eV correspond to C─C and C─CF bonds [44]. Consistent with the XPS results, Fourier‐transform infrared (FTIR) spectroscopy identifies characteristic C─F bending and deformation vibrations at 506.13 and 634.24 cm^−1^, respectively, together with asymmetric and symmetric F─C─F stretching vibrations at 1157.02 and 1214.13 cm^−1^ (Figure 1d) [45]. Energy‐dispersive X‐ray spectroscopy (EDS) further confirms a relatively homogeneous distribution of PTFE across the surface, with a C/F atomic ratio of 1:1.93, close to the theoretical ratio of 1:2 for (C_2_F_4_)_n_ (Figure 1e). Collectively, these spectroscopic and elemental analyses confirm that the prepared surface is dominated by PTFE, with no detectable residual elements from the surfactant or other processing additives.

### 2.2 Effect of Flow Velocity on Icing Behavior Under Shear Flow Condition

Under shear flow conditions, flow velocity governs both the impingement dynamics of microdroplets, including their impact probability and kinetic energy, and the shear stress acting on ice, making it a key parameter in icing evolution. To elucidate the effect of flow velocity on icing behavior, PTFE hydrophobic samples are tested in an icing wind tunnel (Figure 2a). The samples are embedded in the bottom of the observation section to eliminate any height difference between the sample surface and the surrounding wall, thereby minimizing disturbance to the local flow field. Measurements of the microdroplet size distribution within the icing wind tunnel yield a mean droplet diameter of 20.91 µm (Figure 2b), comparable to the characteristic droplet size encountered by aircraft during flight.

**FIGURE 2:**
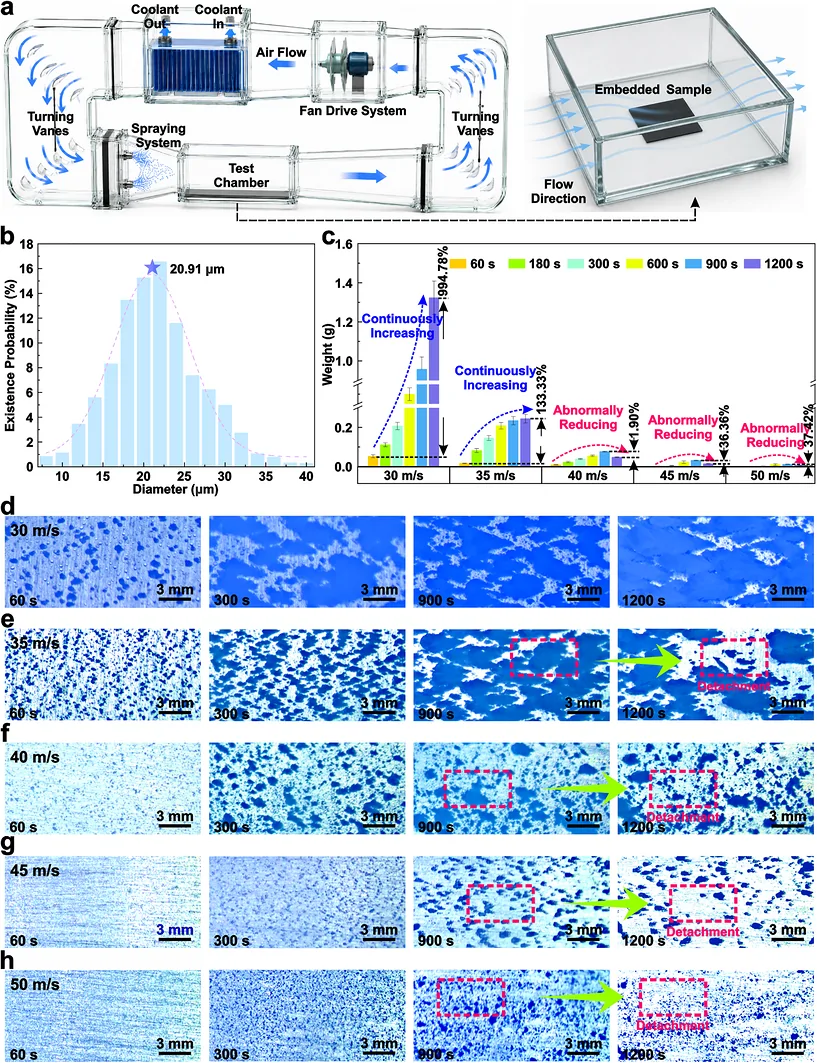
Icing process observation of microdroplets under various flow velocities. (a) Schematic diagram of the device for icing process. (b) Histogram of the diameter distribution of microdroplets. (c) Ice mass on surface under different wind velocities. The repetitive experiments are conducted for five times and data are presented as mean values ± SD. (d) Evolution of ice formation on PTFE‐0 surface at 30 m/s. (e) Evolution of ice formation on PTFE‐0 surface at 35 m/s. (f) Evolution of ice formation on PTFE‐0 surface at 40 m/s. (g) Evolution of ice formation on PTFE‐0 surface at 45 m/s. (h) Evolution of ice formation on PTFE‐0 surface at 50 m/s.

Subsequently, the effect of flow velocity on ice accumulation on the PTFE‐0 surface at ‐15°C is investigated (Figure 2c). At 30 m/s, the accumulated ice mass grows progressively from 0.0078 to 0.0854 g with increasing icing duration, corresponding to an increase of 994.78%. When the flow velocity is fortified to 35 m/s, the ice mass continues to increase monotonically, although the overall growth rate is decreased to 133.33%. Notably, at 40 m/s, the ice mass retained on the PTFE‐0 surface unexpectedly begins to decrease after 900 s of icing, with the mass at 1200 s being 1.90% lower than that at 900 s. This reduction becomes more pronounced at higher wind velocities, reaching 36.36% at 45 m/s and 37.42% at 50 m/s. These results indicate a transition from net ice accumulation to wind‐induced ice shedding as the aerodynamic shear increases.

Under flow conditions, the impinging microdroplets possess finite initial kinetic energy. Assuming that all microdroplets within the airflow impinge on the surface and undergo complete freezing, the maximum theoretical ice accretion rate can be expressed as:

(1) $$\frac{dM}{dt}=\omega \nu A$$

where *ω* denotes the liquid water content of the airflow, ν is the airflow velocity, and A represents the projected cross‐sectional area of the sample normal to the flow direction.

The above relationship indicates that the enlargement of flow velocity increases the theoretical ice accretion rate and hence the accumulated ice mass. To elucidate the unexpected reduction in ice mass observed on PTFE‐0 at higher wind velocities, the icing process is monitored by optical microscopy. It is clear that icing initiates through the coalescence and nucleation of microdroplets at 30 m/s, followed by the growth of protruding ice structures that progressively impede the impingement of subsequent microdroplets. These structures consequently develop a growth advantage and evolve into discrete, feather‐like ice crystals (Figure 2d). Moreover, a similar feather‐like morphology is observed at 35 m/s, but ice detachment also occurs, as indicated by the region highlighted in red in Figure 2e. This means that the ice accretion and detachment occur concurrently, with the accretion rate still exceeding the detachment rate, resulting in a slower net increase in ice mass.

At higher wind velocities, aerodynamic shear progressively disrupts the feather‐like structures, leading to the formation of finer, granular ice morphologies (Figure 2f–h). Moreover, the higher the flow velocity, the smaller the ice crystal size, and the larger the region of ice crystal detachment. Under these conditions, the ice detachment rate exceeds the accretion rate, resulting in a net decrease in retained ice mass. Therefore, it can be inferred that increasing aerodynamic shear progressively shifts the balance from ice growth towards ice detachment, reducing the probability of impinging microdroplets nucleating and remaining on the surface. This conclusion has also been corroborated by icing experiments performed under different flow‐field temperatures (Figure S1).

### 2.3 Ice Evolution Process on Hydrophobic Surface Subjected to Flow Temperature

Although flow velocity strongly influences icing behavior, temperature is a critical parameter governing ice nucleation kinetics and the spatial evolution of ice morphology. Figure 3a shows the icing morphology on PTFE‐0 at 35 m/s after icing for 1200 s under different temperatures. At −5°C, the ice crystals progressively increase in size along the flow direction, evolving from fine granular deposits to a feather‐like structure. Interestingly, an ice‐free region is observed near the leading edge (green box), which may result from the high mobility of microdroplets on the PTFE‐0 surface combined with the delayed nucleation at relatively high temperatures. Afterward, when the temperature is lowered to −10°C, the increased nucleation rate and reduced droplet mobility result in a more uniform distribution of ice‐crystal sizes. Further cooling to −15°C produces a markedly different morphology characterized by the emergence of a large feather‐like ice structure near the leading edge (blue and orange dashed boxes). It is inferred that microdroplets can freeze almost immediately upon impact, giving the leading‐edge region a competitive advantage for ice growth. The resulting protruding crystals subsequently shield downstream regions from incoming droplets, promoting localized and preferential ice growth. At −20°C, extensive ice detachment occurs on the PTFE‐0 surface, indicating that sufficiently strong aerodynamic shear can overcome interfacial retention and induce large‐scale ice detachment. Similar behavior is observed at higher flow velocities Figure S2), further supporting the generality of spontaneous ice detachment from hydrophobic surfaces under shear flow.

**FIGURE 3:**
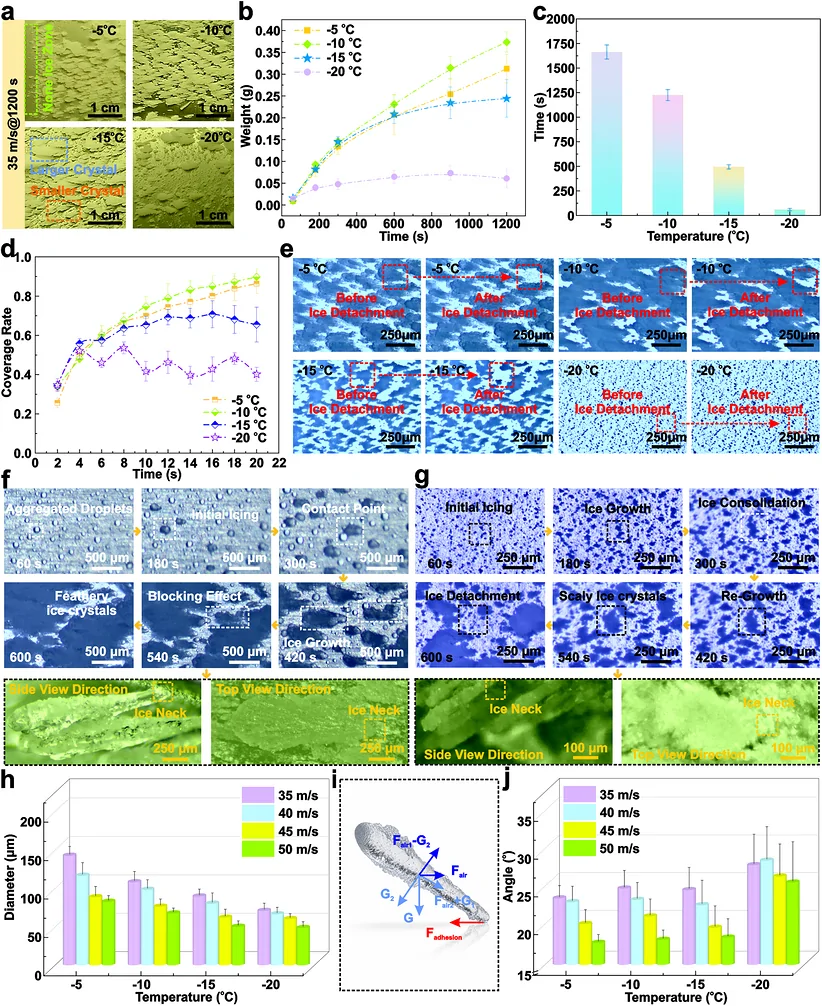
Analysis of dynamic freezing behavior affected by temperature on PTFE‐0 surface. (a) macroscopic morphology of ice crystals with flow velocity of 35 m/s and icing time of 1200 s. (b) Temperature‐dependent evolution of retained ice mass at 35 m/s. (c) Spontaneously detachment time of ice crystals under various temperatures at 35 m/s. (d) Ice coverage under various temperatures at 35 m/s. (e) Surface microscopic morphology before and after the ice detachment under different temperatures at 35 m/s. (f) Typical microscopic evolution process of ice crystal growth at ‐5°C. (g) Typical microscopic evolution process of ice crystal growth at −15°C. (h) Initial ice crystal diameters under different environmental parameters. (i) Schematic diagram of the forces acting on ice crystals in a shear flow field. (j) Orientation angles between ice crystals and substrate under different environmental parameters. All the repetitive experiments are conducted for five times and data are presented as mean values ± SD.

The corresponding evolution of retained ice mass further reveals a strong dependence on temperature (Figure 3b). When the temperature is lower than −10°C, the retained ice mass initially increases with icing duration. Therein, the ice accretion rate is slightly higher than that at −5°C due to the accelerated freezing of droplets upon contact with the surface. However, the net accretion rate is markedly suppressed despite the continued increase in ice mass when the temperature drops to −15°C. At −20°C, the retained ice mass begins to decrease after 15 min of icing, indicating that spontaneous ice fracture and detachment have become sufficiently pronounced to outweigh continued ice accretion. Notably, magnifying the flow velocity further promotes spontaneous ice detachment at low temperatures, expanding the temperature range required for the descent of ice crystal quality. (Figure S3).

To elucidate the role of temperature in ice‐crystal detachment, the onset time of ice detaching from the PTFE‐0 surface is quantified (Figure 3c). It is obvious that the ice coverage enlarges continuously with icing duration at ‐5°C and ‐10°C (Figure 3d). At −15°C, the ice coverage increases rapidly during the initial stage before reaching a relatively stable value, indicating an approximate dynamic balance between ice accretion and detachment. By contrast, the ice coverage exhibits a fluctuating decline, suggesting that ice detachment exceeds continued accretion at −20°C. Examination of the surface morphology before and after ice shedding further reveals that ice crystals detach collectively as discrete units, leaving little discernible residual ice within the corresponding regions of the PTFE‐0 surface (red box in Figure 3e). Particularly, the characteristic size of the detached ice crystals descends with decreasing temperature. Considering that temperature primarily governs the initial droplet nucleation process, it suggests that the morphology established during the initial freezing stage plays an important role in determining the subsequent detachment behavior of ice crystals.

Subsequently, the initial nucleation and freezing dynamics of microdroplets are examined at −5°C. Before the appearance of discernible ice crystals, numerous microdroplets coalesce on the PTFE‐0 surface to form larger droplets (Figure 3f). Nucleation is initiated preferentially on the upstream side of these droplets, followed by the growth of the resulting ice nuclei into protruding crystals, suppressing ice growth downstream. Meanwhile, unfrozen liquid on the ice surface remains sufficiently mobile to redistribute under aerodynamic shear with relatively mild thermal conditions at −5°C, producing ice crystals with feather‐like morphology. Microscopic observations of individual crystals further reveal that these structures are connected to the substrate through a thin ice bridge at an inclined angle. This geometry likely arises from the unidirectional transport of liquid droplets under the combined effects of airflow and gravity, which constrains ice growth predominantly along the droplet‐transport direction. Notably, subsequent enlargement of the feather‐like crystals does not substantially increase their contact area with the substrate, suggesting that the effective ice‐substrate contact area always depends on the impinging droplet size during the initial freezing stage.

At a lower temperature of −15°C, microdroplets freeze directly upon impact with the PTFE‐0 surface, forming granular ice nuclei without an intermediate coalescence stage (Figure 3g). This rapid freezing suppresses the redistribution of unfrozen water over the ice surface, resulting in less regular, flake‐like ice morphologies. Moreover, with continued icing, these initial ice crystals progressively coalesce and grow, significantly increasing the airflow shear force exerted on the overall ice crystals. Since the ice bridges still maintain the connection between the growing crystals and the substrate, it is inferred that the size of the ice crystals governs the aerodynamic loading and hence the critical stress required for spontaneous detachment, whereas the size of the droplets at the initial freezing stage primarily determines the strength of the solid‐ice interfacial bonding.

The initial ice crystal size on the PTFE‐0 surface is statistically evaluated under different thermal and aerodynamic conditions (Figure 3h). Augmenting the flow velocity increases the horizontal momentum of impinging droplets while reducing the number of droplets that effectively contact the surface and subsequently coalesce within a given time interval, thereby suppressing the growth of the initial ice nucleation. Lowering the temperature similarly reduces the initial ice‐crystal size by accelerating freezing and shortening the time available for droplet coalescence, facilitating the detachment of ice crystals. The spontaneous detachment process of ice crystals can be understood as a competition between interfacial adhesion, aerodynamic loading and gravitational force (Figure 3i). Therein, the interfacial adhesion is primarily governed by the initial ice crystal size, whereas the aerodynamic force depends on the flow velocity and the ice crystal morphology, and the gravitational contribution is determined by the overall mass of the ice structure. The dynamic pressure of the airflow can be expressed as follows [46]:

(2) $$F_{air}=P_{d}\times S$$

where *F*_air_ denotes the aerodynamic force exerted by the airflow, *P*_d_ is the corresponding dynamic pressure, and *S* represents the projected frontal area of the ice crystal. The dynamic pressure *P*_d_ can be calculated as follows:

(3) $$P_{d}=\frac{1}{2}\rho v^{2}$$

Here, ρ denotes the air density and v represents the freestream velocity. The net force governing ice‐crystal detachment comprises the gravitational force and the upward component of the aerodynamic force acting on the ice crystal. This resultant force can be expressed as follows:

(4) $$F=F_{air}\times \sin\theta -G\times \cos\theta$$

where *θ* represents the angle between the ice crystal and the substrate.

The inclination of the ice crystal relative to the substrate directly governs both the aerodynamic force acting on the crystal and the gravitational components parallel and normal to the surface. Figure 3j shows the dependence of this inclination angle on the environmental conditions. It is observed that the enlargement of flow velocity enhances the horizontal momentum of impinging microdroplets, reducing their incidence angle, ultimately giving rise to a reduction in the orientation angle of the ice crystal. By contrast, temperature has only a minor effect on the crystal inclination, which remains approximately 22°. In general, although the temperature can reduce the initial size of ice crystals by accelerating the nucleation process, the flow velocity can also increase the shear force acting on the ice crystals by reducing the solid‐ice contact area and increasing the dynamic pressure, which is considered to be a key factor governing the growth and detachment of the ice crystals.

### 2.4 Analysis of Ice Formation Based on Surface Non‐Wetting Differences

Under realistic operating conditions, spontaneous ice detachment is inevitably influenced by the intrinsic physicochemical and structural characteristics of the surface. Differences in surface energy and microtopography alter the contact form of the solid‐ice interface and the balance between ice accretion and detachment. Figure 4a compares the retained ice mass on aluminum alloy (nano‐scale roughness with higher surface energy), PTFE‐0 (nano‐scale roughness with lower surface energy) and PTFE‐10 (micro‐scale roughness with lower surface energy) surfaces after 20 min of icing at 35 m/s under different temperatures. At −5°C, the retained ice mass exceeds 500 mg on both the aluminum alloy and PTFE‐10 surfaces, whereas only 412.6 mg remained on PTFE‐0. With decreasing temperature, the retained ice mass on the aluminum alloy and PTFE‐10 surfaces increases progressively, whereas that on PTFE‐0 reaches a maximum at −10°C before declining markedly at lower temperatures. For the aluminum surface, the higher surface energy strengthens solid‐ice adhesion, thereby favoring continued ice retention as the temperature decreases. In contrast, although the PTFE‐10 surface exhibits substantially lower ice adhesion (Figure S4), its retained ice mass is considerably 15 times higher than that of the PTFE‐0 surface and even exceeds that of the aluminum surface under otherwise identical conditions. Moreover, a higher flow velocity of 50 m/s reduces the retained ice mass on all three surfaces (Figure 4b), while the PTFE‐0 surface consistently exhibits the lower ice retention. Notably, ice accumulation on the PTFE‐10 surface continues to increase with decreasing temperature and remains approximately threefold higher than that on the other two surfaces at −20°C. These results indicate that low interfacial ice adhesion alone does not guarantee efficient ice shedding under sustained aerodynamic shear. Rather, the hierarchical microstructure of the PTFE‐10 surface may mechanically hinder the collective detachment of ice crystals, thereby promoting ice retention despite its intrinsically weak solid‐ice adhesion.

**FIGURE 4:**
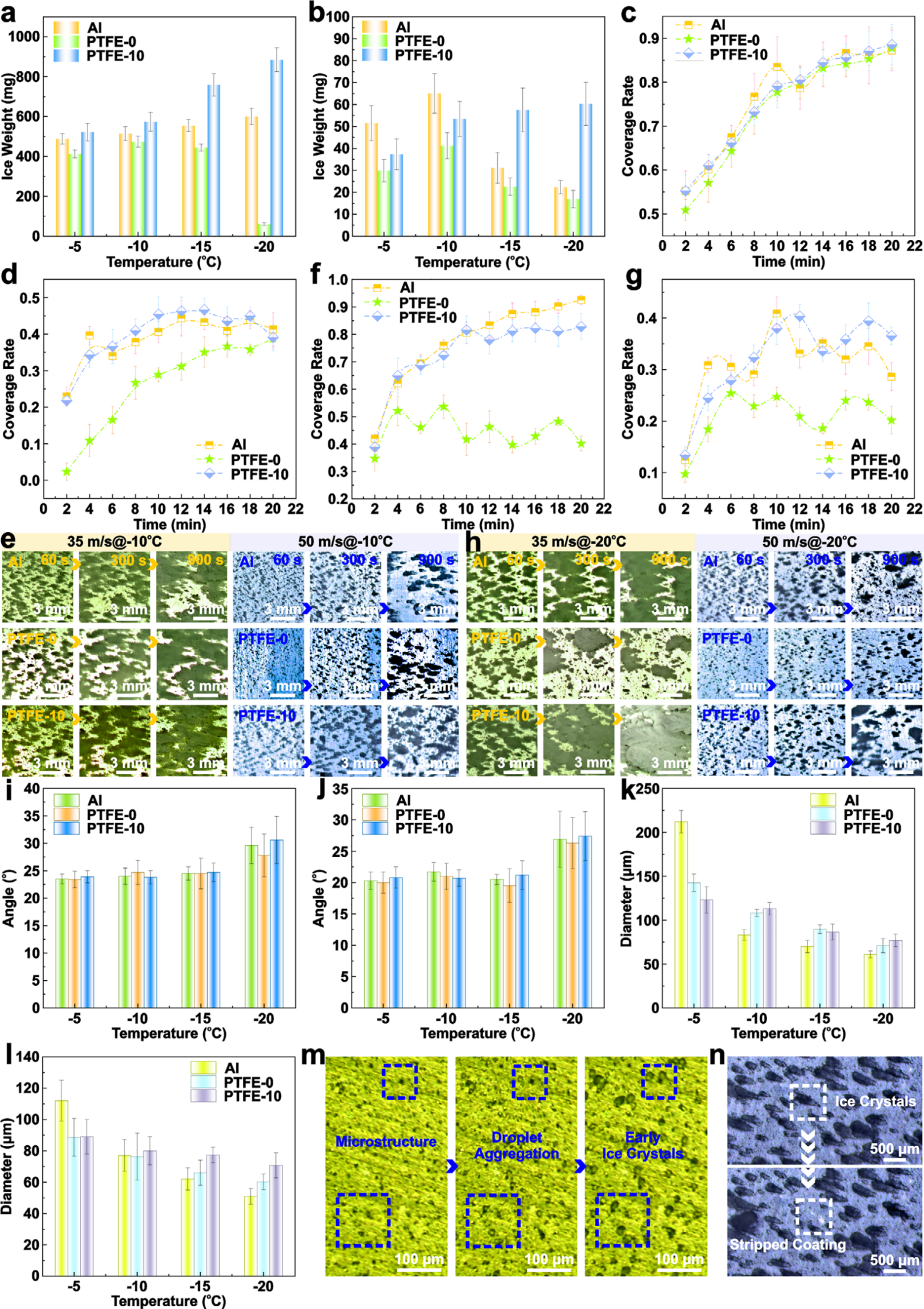
Freezing behavior of surfaces with different wetting properties under shear flow conditions. (a) Retained ice mass on aluminum, PTFE‐0 and PTFE‐10 surfaces after 20 min of icing at 35 m/s under different temperatures. (b) Retained ice mass on aluminum, PTFE‐0 and PTFE‐10 surfaces after 20 min of icing at 50 m/s under different temperatures. (c) Evolution of ice coverage on the three surfaces with icing duration at −10°C with flow velocity of 35 m/s. (d) Evolution of ice coverage on the three surfaces with icing duration at −10°C with flow velocity of 50 m/s. (e) Microscopic morphology of ice accretion on the three surfaces at −10°C with flow velocity of 35 and 50 m/s, respectively. (f) Evolution of ice coverage on the three surfaces with icing duration at −20°C with flow velocity of 35 m/s. (g) Evolution of ice coverage on the three surfaces with icing duration at −20°C with flow velocity of 50 m/s. (h) Microscopic morphology of ice accretion on the three surfaces at −20°C with flow velocity of 35 and 50 m/s, respectively. (i) Inclination angles of ice crystals formed on the three surfaces with flow velocity of 35 m/s. (j) Inclination angles of ice crystals formed on the three surfaces with flow velocity of 50 m/s. (k) Initial ice crystals size formed on the three surfaces with flow velocity of 35 m/s. (l) Initial ice crystals size formed on the three surfaces with flow velocity of 50 m/s. (m) Initial growth and detachment of ice crystals on the PTFE‐10 surface. (n) Microscopic image of localized substrate exposure on the PTFE‐10 surface. All the repetitive experiments are conducted for five times and data are presented as mean values ± SD.

Further analysis is conducted on the evolution of ice coverage on the three surfaces with icing duration under representative conditions at −10°C. At a lower flow velocity of 35 m/s, the onset of ice nucleation is comparable across the three surfaces, followed by a gradual increase in ice coverage with extending icing time (Figure 4c). However, at 50 m/s, the PTFE‐0 surface exhibits a markedly different evolution, with ice coverage rising rapidly throughout the icing process (Figure 4d). On the aluminum and PTFE‐10 surfaces, ice coverage increases rapidly during the initial stage, exceeding 20% within 2 min, and gradually approaching a plateau of approximately 35%. Correlative microscopic observations provide insight into these distinct behaviors (Figure 4e). Under low‐shear conditions, the high thermal conductivity of the aluminum alloy promotes rapid interfacial heat transfer and ice growth, resulting in a higher ice coverage. Whereas the hierarchical microstructure on the PTFE‐10 surface increases the probability of microdroplet retention and surface contact while restricting the migration and detachment of larger droplets, thereby promoting more rapid ice accumulation than on the PTFE‐0 surface. In contrast, the greater mobility of microdroplets on the PTFE‐0 surface suppresses their initial retention and nucleation under high‐shear conditions, favoring the formation of fewer but larger initial ice structures that are subsequently less susceptible to detachment and can continue to grow.

On PTFE‐10 and aluminum surfaces, the microstructure and high surface energy suppress the migration and detachment of deposited droplets, respectively, resulting in rapid initial ice growth. Subsequently, pronounced ice detachment emerges on both surfaces after approximately 4 min, counteracting further increases in ice coverage. This indicates that although aluminum and PTFE‐10 surfaces are more effective at promoting the initial retention and accumulation of microdroplets, the PTFE‐0 surface is more effective at suppressing the persistent retention of runback ice under sustained aerodynamic shear. The same trend is observed under icing conditions at ‐5°C (Figures S5–S8).

At a flow‐field temperature of −20°C, the ice coverage on the PTFE‐0 surface remains approximately 40% under a flow velocity of 35 m/s, while the retained ice mass descends markedly (Figure 4f). In contrast, the ice coverage on both the aluminum and PTFE‐10 surfaces exceeds 80% after 20 min of icing, indicating limited ice detachment during this period. Elevating the flow velocity to 50 m/s induces ice detachment from all three surfaces; nevertheless, PTFE‐0 surface still maintains the lower ice coverage, indicating a greater propensity for its ice crystals to be removed by aerodynamic shear (Figure 4g). The corresponding microscopic imaging reveals extensive ice shedding from PTFE‐0 surface under a lower flow velocity, whereas little discernible detachment occurred on the aluminum and PTFE‐10 surfaces (Figure 4h). Particularly, an exceptionally large ice crystal with a length of approximately 6–8 mm is observed near the leading edge of PTFE‐10 surface, contributing to the anomalously high retained ice mass. Under the higher flow velocity conditions, ice shedding occurs on all three surfaces, but the fraction of ice detached from PTFE‐0 remains substantially higher. These observations are consistent with the results obtained at −15°C (Figures S9–S12), further demonstrating the enhanced susceptibility of the PTFE‐0 surface to wind‐induced ice detachment under severe icing conditions.

To elucidate the origin of the anomalous ice growth on the PTFE‐10 surface, the inclination angles of ice crystals formed on the three surfaces under different wind velocities are analyzed, as demonstrated in Figure 4i,j. Across the investigated environmental conditions, the crystal angles show no substantial differences among the surfaces, indicating that this parameter is governed predominantly by the external flow and thermal conditions, with only a limited contribution from surface physicochemical properties. The statistics of initial ice crystal size indicate that the higher wettability of the aluminum alloy promotes droplet spreading and reduces the extent of droplet coalescence before freezing under low‐wind‐velocity conditions. As the temperature decreases, the shortened coalescence time causes a larger fraction of microdroplets to freeze before sufficient aggregation, leading to a pronounced reduction of the initial ice‐crystal size from 212 to 61 µm (Figure 4k). Consequently, the initial ice crystals on the aluminum surface remain substantially smaller than those on both PTFE surfaces.

Additionally, the two PTFE surfaces exhibit comparable initial ice‐crystal sizes under low‐wind‐velocity conditions. Given that the characteristic size of the microdroplets is comparable to the surface texture of PTFE‐10, microdroplets can penetrate into the microstructure, destabilizing the Cassie‐Baxter wetting state and increasing their effective solid‐liquid contact. The resulting interfacial contact configuration is therefore similar to that on PTFE‐0, which may account for the comparable initial ice‐crystal sizes of the two surfaces. Subsequently, augmenting the flow velocity further reduces the difference in initial ice‐crystal size among the three surfaces (Figure 4l). Notably, the microstructure of the PTFE‐10 surface increasingly restricts droplet migration and promotes droplet retention at −15°C, leading to a larger initial ice crystal, an enhanced ice accumulation and a higher net icing rate.

However, irrespective of flow velocity, the initial ice‐crystal sizes on PTFE‐0 and PTFE‐10 surfaces differ by less than 10%, indicating that the initial crystal size alone cannot account for the pronounced anomalous ice growth on the PTFE‐10 surface. The icing process reveals that the microstructure of the PTFE‐10 surface promotes droplet retention within the microstructure regions, where droplets preferentially accumulate and freeze (blue box in Figure 4m). The resulting ice nuclei can completely envelop the underlying microstructures, establishing strong mechanical anchoring at the solid‐ice interface. During subsequent aerodynamic loading, detachment of ice crystals requires overcoming not only interfacial adhesion but also the mechanical constraint imposed by the embedded microstructures, thereby substantially improving the critical shear stress for ice shedding. Particularly, the enhanced anchoring is sufficiently strong to cause simultaneous delamination of the PTFE coating from the underlying substrate during ice removal, even exposing the substrate (Figure 4n). Therefore, it is considered that droplet retention and mechanical anchoring induced by microstructure are the key contributors to the enhanced effective solid‐ice adhesion of the superhydrophobic surface under shear‐flow conditions. This anchoring effect promotes the persistence and subsequent growth of individual ice crystals, ultimately giving rise to the anomalous ice accumulation observed on the PTFE‐10 surface.

### 2.5 Diverse Ice Repulsion Behavior Induced by Structural Scales

Considering the pronounced influence of the microstructure on the solid‐ice adhesion, the ice crystal retention mass of PTFE surfaces with different microstructure scales is analyzed under typical temperature conditions to explore the structural scale suitable for the anti‐icing function. It is clear that the retained ice mass increases progressively from 36.3 to 189.7 mg with rising structural scale at −5°C (Figure 5a). Based on the previous conclusions, the microstructures impede droplet migration, promoting droplet accumulation and initial freezing within the microstructure regions. Elevating the structural scale intensifies this confinement and provides more favorable sites for ice nucleation, thereby promoting ice accumulation. Consistent with this mechanism, ice coverage improves systematically with microstructural scale (Figure 5b). Although the ice coverage on all surfaces approached a relatively slow‐growth regime after approximately 4 min, only the PTFE‐0 surface exhibits a significant decrease after 18 min, whereas ice continued to accumulate on the microstructured PTFE surfaces.

**FIGURE 5:**
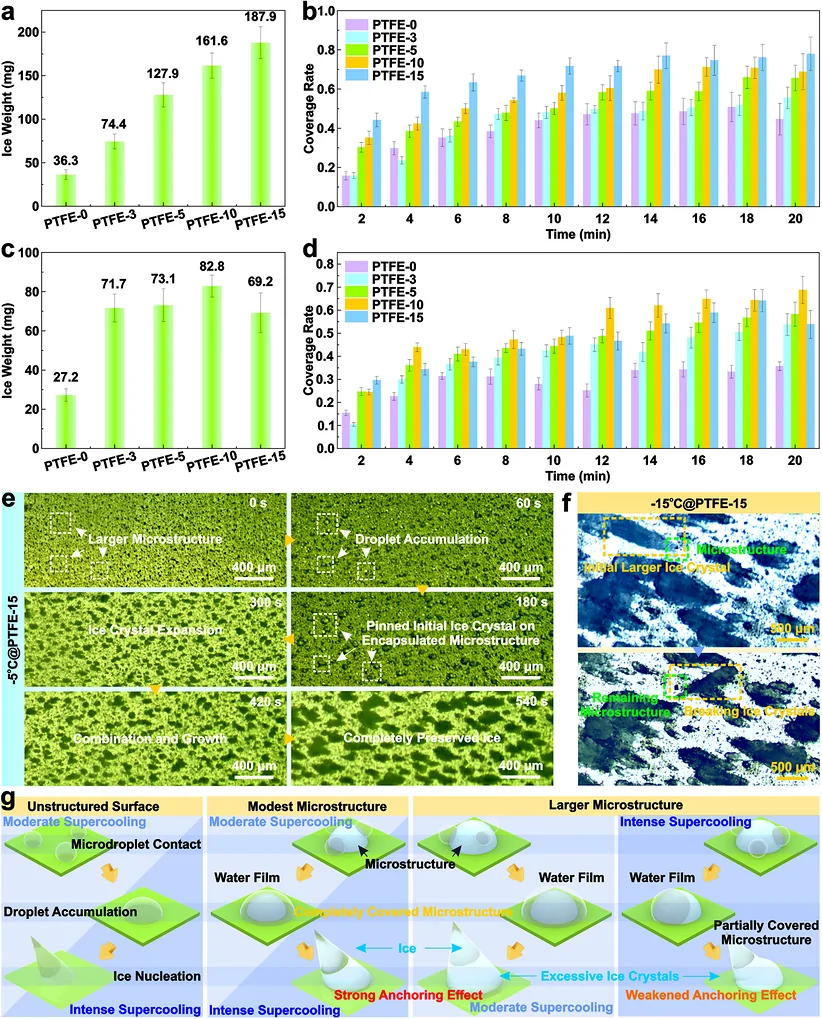
Icing behavior of PTFE surfaces with various microstructural scales under shear flow conditions. (a) Retained ice mass on different PTFE surfaces after 20 min of icing at 35 m/s under a temperature of −5°C. (b) Evolution of ice coverage rate on different PTFE surfaces at 35 m/s under a temperature of −5°C. (c) Retained ice mass on different PTFE surfaces after 20 min of icing at 35 m/s under −15°C. (d) Evolution of ice coverage rate on different PTFE surfaces at 35 m/s under a temperature of −15°C. (e) Microscopic morphology of ice growth and detachment on the PTFE‐15 surface at 35 m/s under a temperature of −5°C. (f) Typical ice detachment behavior on PTFE‐15 surface at 35 m/s under a temperature of −15°C. (g) Mechanism of microstructure‐mediated ice fracture under shear flow. All the repetitive experiments are conducted for five times and data are presented as mean values ± SD.

At −15°C, the retained ice mass on all micro‐structured PTFE surfaces remains substantially higher than that on the PTFE‐0 surface (Figure 5c). However, further enlargement of the structural scale no longer results in incremental ice accumulation, with the retained mass remaining approximately 75 mg or even decreasing on surfaces with the larger structures. Notably, the PTFE‐0 surface maintains the lower ice coverage, whereas the four micro‐structured PTFE surfaces exhibit comparatively similar coverage (Figure 5d). Particularly, the PTFE‐15 surface with a higher microstructure scale no longer shows the higher ice coverage and instead exhibits a decrease after approximately 18 min, indicating pronounced ice detachment. It suggests that decreasing temperature alters the balance between microstructure‐mediated ice retention and aerodynamic detachment, progressively weakening the anchoring effect of the microstructures and facilitating ice shedding at larger structural scales. The influence of structural scale on surface ice crystal retention behavior under different temperature conditions is also verified by the evolution of critical flow velocity (Figures S13 and S14).

Further examination of ice‐crystal evolution on the PTFE‐15 surface demonstrates a pronounced dependence of microstructure‐mediated ice anchoring on temperature. The droplet accumulation persists for an extended period at −5°C, with microdroplets progressively accumulating around the surface microstructures until they are almost completely encapsulated before freezing into initial ice crystals (Figure 5e). Once the structures are fully embedded within the ice layer, subsequent aerodynamic forcing produces relative motion within the ice but rarely results in complete detachment from the substrate. The ice crystal coverage rate is governed primarily by the number of microdroplets contacting the sample surface. The larger the surface structure scale, the more microdroplets contact the surface, and the greater the growth rate of ice crystal coverage.

However, the shortened droplet‐aggregation time prevents complete encapsulation of the larger microstructures when the temperature decreases to −15°C. Instead, microdroplets preferentially accumulate and freeze on the windward side of individual microstructures, leaving part of the microstructure exposed and substantially reducing mechanical anchoring, resulting in a lower critical driving stress for aerodynamic detachment (red box in Figure 5f). Hence, it is concluded that the mechanical anchoring induced by the microstructure is a key determinant of the effective solid‐ice adhesion of superhydrophobic surfaces. Critically, the anchoring effect reaches its maximum only when the ice‐crystal root fully envelops the underlying microstructure. When droplet aggregation is kinetically limited or the microstructural scale becomes sufficiently large to prevent complete encapsulation, this anchoring effect is weakened, allowing ice crystals to detach even under comparatively moderate aerodynamic shear.

In general, hydrophobic coatings with nanostructure combining low droplet adhesion, pronounced non‐wettability and low thermal conductivity can suppress initial droplet freezing under shear‐flow conditions, facilitating the separation of droplets and the spontaneous fracture of accreted ice crystals. Conversely, a superhydrophobic surface with microscale topography always weakens icephobic performance by restricting microdroplet migration, promoting droplet accumulation within the microstructure regions. Subsequent encapsulation of the microstructures by nascent ice crystals establishes strong mechanical anchoring at the solid‐ice interface, accelerating abnormal ice growth that further suppresses ice detachment. The relevant mechanism is schematically illustrated in Figure 5g. Therefore, it demonstrates that nanoscale surface structures can offer a distinct advantage over hierarchical micro‐nanostructures in suppressing the retention of runback ice under sustained aerodynamic shear.

## 3 Conclusions

In this work, the evolution of runback ice on PTFE surfaces with different wetting characteristics was investigated under shear flow conditions. It was observed that an external flow field fundamentally altered ice adhesion dynamics by inducing the spontaneous fracture and detachment of ice crystals through aerodynamic shear forces. Interestingly, the effective ice‐substrate contact area was primarily governed by the initial droplet size at the onset of freezing, determining the interfacial adhesion strength between the ice crystals and the substrate. Notably, lower temperatures shortened the droplet‐accumulation period and reduced the size of the initial ice crystals, facilitating subsequent ice detachment. Likewise, higher flow velocity also increased the horizontal velocity of microdroplets, reducing their residence and accumulation time and consequently decreasing the initial ice‐crystal size. Thus, spontaneous ice detachment under shear flow was dominated by the competition between the interfacial adhesion associated with initial ice crystals and the aerodynamic force resulting from their orientation angle.

Particularly, a higher surface energy promoted droplet adhesion and enhanced microdroplet retention, accelerating ice growth and ascending the retained ice mass. The stronger solid‐ice interfacial bonding associated with high surface energy further raised the critical stress required for spontaneous ice detachment. However, superhydrophobic surfaces even exhibited substantially greater ice retention than hydrophobic surfaces with comparable surface energy. This counterintuitive behavior primarily arose from mechanical anchoring between the surface microstructures and the ice crystals, which enlarged the resistance to complete ice detachment. Moreover, the microstructures could become fully encapsulated by the accumulated droplets and subsequent ice when the droplet aggregation stage was prolonged, intensifying the anchoring effect and potentially promoting the formation of anomalously large ice crystals.

In general, increasing the characteristic structural scale strengthened the mechanical anchoring and consequently suppressed complete ice detachment. Although a lower temperature shortened the droplet‐accumulation period and left larger microstructures only partially encapsulated, the resulting interfacial anchoring could still remain substantially stronger than that on an unstructured hydrophobic surface, increasing the critical stress for spontaneous ice detachment. These findings demonstrated that reducing the characteristic structural scale of a superhydrophobic surface could be more advantageous than adopting conventional hierarchical micro‐nanostructures for suppressing runback ice under shear flow conditions, highlighting the importance of weakening ice anchoring induced by structural size in the design of icephobic surfaces.

## 4 Experimental Section/Methods

### 4.1 Preparation and Characterization of PTFE Surfaces

A 2 cm × 2 cm × 2 mm 2024 aluminum alloy substrate was ground and polished, followed by sequential ultrasonic cleaning in acetone, anhydrous ethanol, and deionized water for 30 min each. After drying, 0.08 mL of a PTFE nanoparticle suspension containing a non‐ionic surfactant was uniformly spray‐coated onto the substrate to obtain the PTFE‐0 precursor. After natural drying, 3, 5, 10, and 15 mL of the PTFE nanoparticle suspension were subsequently spray‐coated onto the PTFE‐0 surface precursor to fabricate the PTFE‐3, PTFE‐5, PTFE‐10, and PTFE‐15 precursors, respectively. The resulting samples were thermally cured in a muffle furnace by heating at 5°C/min to 360°C, maintaining this temperature for 1 h, and then water‐cooling to obtain the final coatings.

The surface morphology and three‐dimensional topography were characterized by field‐emission scanning electron microscopy and confocal microscopy, respectively. Static water‐contact‐angle measurements were performed to evaluate surface wettability, while X‐ray photoelectron spectroscopy (XPS), Fourier‐transform infrared spectroscopy (FTIR) and energy‐dispersive X‐ray spectroscopy (EDS) were used to determine the surface chemical composition. Detailed information on the reagents, equipment and experimental procedures is provided in the Supplementary Information.

### 4.2 Icing Process of Microdroplets Under Shear Flow Conditions

To eliminate disturbance of the airflow by the sample geometry and avoid its influence on ice‐crystal growth, the sample was flush‐mounted in the bottom wall of the wind‐tunnel observation section. The size distribution of the microdroplets was characterized using a custom‐built laser detection system. The outlet of the microdroplet generator was aligned with the laser beam, and individual droplet diameters were determined from the intensity of the reflected laser signal. The droplet‐size distribution was then obtained by statistically analyzing the measured droplet diameters and corresponding number counts. To verify the spatial uniformity of the microdroplet field, an additional grid‐based icing test was performed, confirming a homogeneous droplet distribution throughout the wind‐tunnel test section (Figure S15).

### 4.3 Icing Morphology Observation of Microdroplets in Flow Environment

The observation section of the ice wind tunnel was constructed from transparent acrylic to enable direct optical access. A stereo microscope and a CCD camera were positioned above the observation section to resolve the microscopic morphology of surface ice and capture its temporal evolution during icing. The photographs of the experimental setup are provided in Figure S16. Additionally, a high‐speed camera operating at up to 200 000 frames s‐1 was employed to resolve the initial nucleation of ice crystals and their subsequent spontaneous detachment under different incoming‐flow conditions.

### 4.4 Statistical Data on the Freezing of Microdroplets Under Flow Conditions

The retained ice mass was determined from the difference in sample mass before and after icing using an analytical balance. Ice coverage was quantified by a non‐contact image‐analysis method based on optical micrographs acquired with a stereomicroscope. The ice layer and underlying substrate were distinguished by adjusting image brightness and contrast, and the ice‐covered area was subsequently extracted by image processing. For each sample, five randomly selected regions were analyzed, and the mean value was used to minimize local heterogeneity and measurement uncertainty. The inclination angle of the ice crystals relative to the substrate was also determined by stereomicroscopy. After icing, the microscope was repositioned laterally with its optical axis parallel to the sample surface, enabling direct measurement of the angle between the principal axis of individual ice crystals and the substrate. Five spatially separated ice crystals were selected for each sample, and the mean inclination angle was calculated.

## Author Contributions

**Jiawei Jiang**: Writing – original draft, conceptualization, methodology, investigation, formal analysis. **Yizhou Shen**: writing – review and editing, funding acquisition, supervision, conceptualization. **Yangjiangshan Xu**: methodology, software, data curation, validation. **Junxian Xiang**: conceptualization, validation. **Weilan Liu**: validation, data curation. **Senyun Liu**: visualization, software. **Lingfeng Zhao**: validation, investigation. **Jie Tao**: writing – review and editing, supervision. **Zhong Chen**: supervision, writing – review and editing.

## Conflicts of Interest

The authors declare no conflicts of interest.

## Supporting information




**Supporting File**: advs78489‐sup‐0001‐SuppMat.docx.

## Data Availability

The data that supports the findings of the study are available from the corresponding author.
