# Supplementary material for: Enhancing Surface Icephobic Performance via Spontaneous Fracture Characteristics of Ice Crystals Regulated by Structural Scale Under Shear Flow Conditions
Source: Adv Sci (Weinh). 2026 Oct 25:e78489. Online ahead of print. doi: 10.1002/advs.78489 (PMC13696531; doi:10.1002/advs.78489)
Supplement: Supplementary file 1 [file ADVS-9999-e78489-s001.docx]

Supporting Information

Enhancing surface icephobic performance via spontaneous fracture characteristics of ice crystals regulated by structural scale under shear flow conditions

Jiawei Jiang^1^, Yizhou Shen^1*^, Yangjiangshan Xu^2^, Junxian Xiang^1*^, Weilan Liu^3^, Senyun Liu^4^, Lingfeng Zhao^1^, Jie Tao^1^, Zhong Chen^5^

1. Results and Discussion

1.1. Effect of Wind Velocity on Icing Behavior under Shear Flow Condition

At -5°C, ice accretion on PTFE-0 proceeds at all investigated wind velocities through the nucleation and freezing of coalesced droplets. Initially, accumulated droplets undergo localized nucleation and develop into protruding ice structures that shield the underlying surface from subsequent droplets, thereby promoting preferential growth and ultimately producing discrete feather-like crystals (**Figure S1**).

**Figure S1.** Microscopic growth process of ice crystals under different wind velocities at -5 ℃

Increasing the flow velocity progressively alters this growth mode. Below 40 m/s, feather-like crystals develop without appreciable shedding, whereas the morphology transitioned towards slender, rod-like structures at 40 m/s. At 45 m/s and 50 m/s, droplet accumulation is markedly suppressed, delaying the onset of visible ice formation to 298 s and 811 s, respectively. The resulting crystals are substantially smaller and evolved from the characteristic narrow-base, broad-tip morphology to compact rod-like structures, accompanied by pronounced spontaneous ice shedding.

1.2. Ice Evolution Process on Hydrophobic Surface Subjected to Flow Temperature

At a flow velocity of 50 m/s and a temperature of -5°C, portions of the leading edge remain free of ice despite prolonged icing (**Figure S2**). As the temperature decreases, the ice crystals formed on the PTFE surface become progressively smaller and less numerous. At -20°C, ice retention is further suppressed, with substantially fewer residual crystals and extensive spontaneous ice shedding from the surface.

**Figure S2.** Macroscopic morphology of ice crystals at different temperatures when the wind velocity is 50 m/s.

The ice-mass accumulation is markedly suppressed across the investigated temperature range with a flow velocity of 50 m/s (**Figure S3**). At -5°C, the retained ice mass remains nearly unchanged during the first 10 min of icing, while only a modest increase is observed at -10°C. Upon extending the icing duration to 15 min, rapid ice accumulation emerges at both -5°C and -10°C, whereas the retained ice mass decreases sharply at -15°C and -20°C, indicating pronounced ice detachment. Compared with the 35 m/s condition, the higher flow velocity promotes aerodynamic ice removal at lower temperatures, thereby extending the temperature range over which the retained ice mass decreases.

**Figure S3.** Changes in ice crystal mass under a wind velocity of 50 m/s.

1.3. Analysis of Ice Formation Based on Surface Non-Wetting Differences

Compared with the aluminum alloy substrate, the PTFE surfaces substantially reduce ice adhesion (**Figure S4**). Notably, introducing microstructures further decreases the ice adhesion strength of PTFE-10 to 32.16 kPa, corresponding to 53.6% of that measured for the PTFE-0 surface and only 15.37% of that for the aluminum alloy surface. Despite its pronounced non-wettability, PTFE-10 therefore exhibits exceptionally weak static ice adhesion, highlighting its strong potential for passive ice-repellent applications.

**Figure S4.** Adhesion forces of ice layers on different material surfaces.

**Figure S5** shows that ice coverage on all three surfaces ascends progressively with icing time with a flow velocity of 35 m/s at -5°C. Few appreciable ice detachments are observed during the observation period.

**Figure S5.** Changes in surface ice crystal coverage with a flow velocity of 35 m/s at -5°C.

The higher thermal conductivity of the aluminum alloy promotes more rapid ice growth, yielding slightly greater ice coverage than that of the PTFE-0 surface (**Figure S6**). The PTFE-10 surface exhibits a markedly higher initial ice-growth rate, which can be attributed to its microstructure: the surface features increase the probability of droplet-substrate contact while suppressing droplet migration and detachment, thereby enhancing droplet retention and promoting ice accumulation.

**Figure S6**. Changes in the microscopic morphology of three types of surface ice crystals under a temperature of -5℃ and a wind velocity of 35 m/s.

At the flow velocity of 50 m/s, ice coverage on the aluminum alloy initially increases but subsequently declines, whereas both PTFE surfaces exhibit a continued enlargement in ice coverage (**Figure S7**). Microscopic observations indicate that the strong droplet adhesion on aluminum alloy suppresses droplet migration, allowing droplets to remain on the surface even under high aerodynamic shear (**Figure S8**). Rapid ice growth subsequently augments the aerodynamic loading on larger crystals, triggering partial shedding after 10 min and thereby reducing the overall ice coverage. By contrast, the high droplet mobility on PTFE promotes droplet removal under strong airflow, delaying ice accumulation and yielding smaller ice crystals during the early icing stage. Notably, the PTFE-0 surface exhibits more extensive ice shedding than PTFE-10, indicating that the hydrophobic, nanoscale-textured surface provides superior ice-repellent performance under high-shear flow than the hierarchical superhydrophobic surface.

**Figure S7.** Changes in the coverage of surface ice crystals under -5℃ and 50 m/s wind conditions.

**Figure S8.** Microscopic morphological changes of three types of surface ice crystals under a wind velocity of 50 m/s.

When the temperature further drops to -15℃, the influence of the high thermal conductivity on the freezing behavior of microdroplets on the aluminum alloy surface is weakened under a lower flow velocity, resulting in comparable initial crystallization times across the three surfaces and progressive increases in ice coverage (**Figure S9**). However, ice coverage on PTFE-10 approaches a plateau after 10 min, whereas it continues to increase on aluminum alloy surface and PTFE-0 surface, ultimately leaving PTFE-10 surface with the lower overall coverage.

**Figure S9.** Changes in the coverage of surface ice crystals under -15℃ and 35 m/s wind conditions.

Corresponding microscopic observations reveal that a small number of anomalously large ice crystals with lengths up to ~6 mm is concentrated near the windward edge of the PTFE-10 surface (**Figure S10**). These crystals obstruct subsequent microdroplet impingement and suppress downstream ice growth, such that the accumulated ice mass is dominated by a few large crystals. This behavior further indicates strong interfacial anchoring on the PTFE-10 surface, which cannot be explained solely by the initial droplet size and instead arises from the interaction between the ice and the surface microstructure.

Moreover, appreciable ice shedding from the aluminum alloy and PTFE-0 surfaces commences after approximately 6 min of icing at a flow velocity of 50 m/s, whereas detachment from the PTFE-10 surface is delayed, with its ice-coverage growth rate declining only after about 8 min (**Figure S11**). Consequently, the PTFE-10 surface retains substantially greater ice coverage. The enhanced aerodynamic loading also suppresses the formation of anomalously large ice crystals on the PTFE-10 surface (**Figure S12**). Nevertheless, although ice detachment occurs on all three surfaces under high-shear conditions, the PTFE-0 surface still exhibits the higher spontaneous detachment fraction, further demonstrating its superior icephobic performance.

**Figure S10.** Changes in the microscopic morphology of three types of surface ice crystals with wind velocity of 35 m/s conditions at -15℃.

**Figure S11.** Changes in surface ice crystal coverage with wind velocity of 50 m/s conditions at -15℃.

**Figure S12.** Changes in the microscopic morphology of three types of surface ice crystals with wind velocity of 50 m/s conditions at -15℃.

**1.4. Diverse Ice Repulsion Behavior Induced by Structural Scales**

The variation of flow velocity at the critical fracture of ice crystals on PTFE surfaces with different structural scales are also investigated (**Figure S13**). The results show that when the temperature is -5℃, as the surface structural scale increases, the critical flow velocity required for the fracture of ice crystals gradually increases from 15 m/s on the PTFE-0 surface to 55 m/s on the PTFE-15 surface. This is highly consistent with the evolution process of ice mass on the surface area.

**Figure S13.** The influence of structural scale on the critical flow velocity at -5℃.

However, at -15°C, although the critical flow velocity generally increased with structural scale, its magnitude was substantially lower than that at -5°C (**Figure S14**). The lower temperature accelerates the initial freezing of water droplets, resulting in smaller nascent ice crystals that are more readily fractured and detached by aerodynamic shear. At the largest structural scale, the anchoring effect is instead weakened, lowering the initiation stress for spontaneous ice-crystal detachment. Consequently, the critical wind speed of PTFE-15 falls to 35 m/s, highlighting the coupled effects of initial crystal size and structural anchoring on shear-induced ice shedding. This is consistent with the conclusions obtained in Figures 5c and 5d of the main text.

**Figure S14.** The influence of structural scale on the critical flow velocity at -15℃.

2. Experimental Section/Methods

*Preparation and Characterization of PTFE Surfaces*

The 2024 aluminum alloy was sourced from Southwest Aluminum Industry. PTFE dispersion (60%) was obtained from Shanghai Aladdin Biochemical Technology Co., Ltd., while anhydrous ethanol (AR) and acetone (AR) were supplied by National Pharmaceutical Group Chemical Reagent Co., Ltd. Deionized water was prepared in-house using a Ulupure-II-20T purification system.

The aluminum alloy was cut into specimens measuring 20 mm × 20 mm × 2 mm. Surface oxides were removed sequentially using 400#, 800#, 1000#, 1500#, and 2000# metallographic sandpapers, followed by polishing to a mirror finish. Samples were cleaned ultrasonically in acetone, anhydrous ethanol, and deionized water (15 min each), then dried at 80 °C for 2 h before storage.

The 0.08 ml of PTFE nanoparticle dispersion containing non-ionic surfactant was sprayed onto the cleaned aluminum substrate at a pressure of 0.4 MP from a distance of 80 cm to prepare the PTFE-0 sample. After air-drying in a fume hood for 12 h, additional PTFE dispersions (3 ml, 5 ml, 10 ml, and 15 ml) were sprayed onto the pre-prepared PTFE-0 using the same procedure to obtain PTFE-3, PTFE-5, PTFE-10, and PTFE-15 samples, respectively. All samples were heat-treated in a muffle furnace at 5 °C/min to 360 °C, held for 1 h, and then quenched in room temperature water.

Surface morphology and roughness were characterized using a field-emission scanning electron microscope (FE-SEM, Hitachi S4800, Japan) and an optical profilometer (Bruker Contour GT-K 3D, Germany). Chemical composition was analyzed by Fourier transform infrared spectroscopy (FTIR, Nicolet IN10, ThermoFisher), X-ray photoelectron spectroscopy (XPS, Thermo Scientific K-Alpha), and energy-dispersive X-ray spectroscopy (EDS). Surface wettability was evaluated using a contact angle goniometer (Kruss DSA100, Germany).

*Icing process of microdroplets under shear flow conditions*

Grid test results indicated that the ice surface area was relatively uniform, demonstrating the homogeneity of droplet distribution within the ice wind tunnel, as presented in **Figure S15a**. Additionally, the sample is embedded in the wall at the bottom of the observation section in order to prevent the shape of the sample from disturbing the flow field and affecting the growth of ice crystals on the surface in the wind tunnel, as demonstrated in **Figure S15b**.

**Figure S15.** Design and verification of wind tunnel ice formation test: a grid test in ice wind tunnel. b Placement method of sample.

*Icing Morphology Observation of Microdroplets in Flow Environment*

A vertical circulating ice wind tunnel was used to simulate the icing behavior on the sample during real flight, as shown in **Figure S16**. The flow velocity range of the ice wind tunnel was from 0 m/s to 70/m s, and the temperature range was from room temperature to -30oC. The diameter of microdroplets was regulated within the range of 10 μm~40 μm by adjusting the air and water pressures in the droplet generation system. The observation section of the wind tunnel is transparent to facilitate subsequent observation of the sample under high-velocity fluid conditions.

**Figure S16**. The test ice wind tunnel used in our work.
